# Supplementary material for: New genomic data and analyses challenge the traditional vision of animal epithelium evolution
Source: BMC Genomics. 2018 May 24;19:393. doi: 10.1186/s12864-018-4715-9 (PMC5968619; doi:10.1186/s12864-018-4715-9)
Supplement: Supplementary file 1 — Figure S1A. Comparison of p120 sequences. Residues involved in interaction with E-cadherin are boxed in red. Most of them are conserved. Figure S1B. Comparison of β-catenin sequences. A single β-catenin gene copy was identified in every studied species except for calcareous sponges that exhibit a duplication. All residues essential for E-cadherin interaction are boxed in pink and are highly conserved except for the R386 and N387 residues (replaced by L and T, respectively) in two hexactinellids and a more anecdotal change from A656 to S in placozoans. Residues boxed in blue are involved in α-catenin binding and in orange for the DTDL PDZ binding motif. Figure S1C. Analyses of α-catenins and vinculins sequences. Sequences of α-catenins and vinculins were aligned based on the structural domains helix0 to helix5 in Mus musculus α-catenin and vinculin. Helices are boxed and the numbers at the end of each sequence indicate the range encompassed in the alignment. Secondary structure prediction by JNet (Jalview option) identified six helices in all sponge α-catenin sequences except for A. queenslandica (missing the 4 first helices) and A. vastus (missing helix0). All species analyzed in this study have one copy of α-catenin and one copy of vinculin well-separated in Bayesian tree with high support (pp = 1) (bottom). Figure S2. Structure of Par3 proteins in metazoans. Par3 exhibits a conserved N-terminal domain (CR1), three central PDZ domains, and a C-terminal region containing multiple protein binding sites including the aPKC-binding motif. Figure S5. Domain composition of PatJ (D. melanogaster), INADL and MUPP1 (M. musculus) and Multiple PDZ containing protein (MPDZ) (O. lobularis, S. ciliatum, A. queenslandica and O. minuta). Note that only O. lobularis exhibits an MPDZ with a well-detected L27 domain (Evalue = 8.5 10− 4) as bilaterians. A. queenslandica and S. ciliatum MPDZ have a low-scoring L27 domain (shaded in grey) according to the HMM profile search. There is no [file 12864_2018_4715_MOESM1_ESM.pdf]

Figure S1A. **Comparison of p120 sequences**. Residues involved in interaction with E-cadherin are boxed in red. Most of them are conserved.

Figure S1B. **Comparison of β-catenin sequences**. A single β-catenin gene copy was identified in every studied species except for calcareous sponges that exhibit a duplication. All residues essential for E-cadherin interaction are boxed in pink and are highly conserved except for the R386 and N387 residues (replaced by L and T, respectively) in two hexactinellids and a more anecdotal change from A656 to S in placozoans. Residues boxed in blue are involved in α-catenin binding and in orange for the DTDL PDZ binding motif.

Figure S1C. **Analyses of α-catenins and vinculins sequences**. Sequences of α-catenins and vinculins were aligned based on the structural domains helix0 to helix5 in *Mus musculus* α-catenin and vinculin. Helices are boxed and the numbers at the end of each sequence indicate the range encompassed in the alignment. Secondary structure prediction by JNet (Jalview option) identified six helices in all sponge α-catenin sequences except for *A. queenslandica* (missing the 4 first helices) and *A. vastus* (missing helix0). All species analyzed in this study have one copy of α-catenin and one copy of vinculin well-separated in Bayesian tree with high support (pp=1) (bottom).

Figure S2. **Structure of Par3 proteins in metazoans**. Par3 exhibits a conserved N-terminal domain (CR1), three central PDZ domains, and a C-terminal region containing multiple protein binding sites including the aPKC-binding motif.

Figure S5. Domain composition of PatJ (*D. melanogaster*), INADL and MUPP1 (*M. musculus*) and Multiple PDZ containing protein (MPDZ) (*O. lobularis, S. ciliatum, A. queenslandica* and *O. minuta*). Note that only *O. lobularis* exhibits an MPDZ with a well-detected L27 domain (Evalue=8.5 10^{-4}) as bilaterians. *A. queenslandica and S. ciliatum* MPDZ have a low-scoring L27 domain (shaded in grey) according to the HMM profile search. There is no recognizable similarity to the L27 domain in the N-terminal region *of O. minuta* MPDZ.

Belahbib et al. Figure S1A

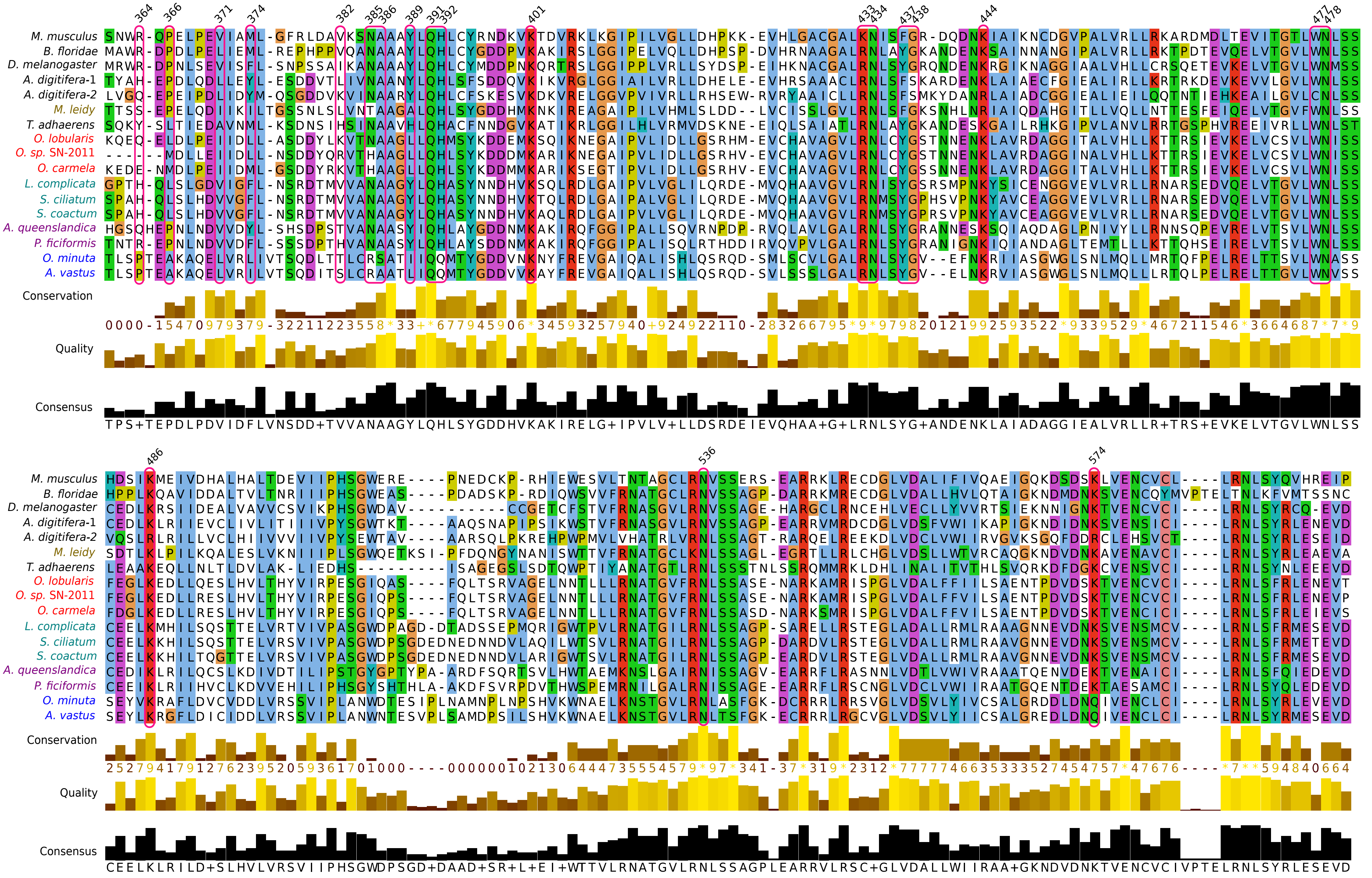

Belahbib et al. Figure S1B

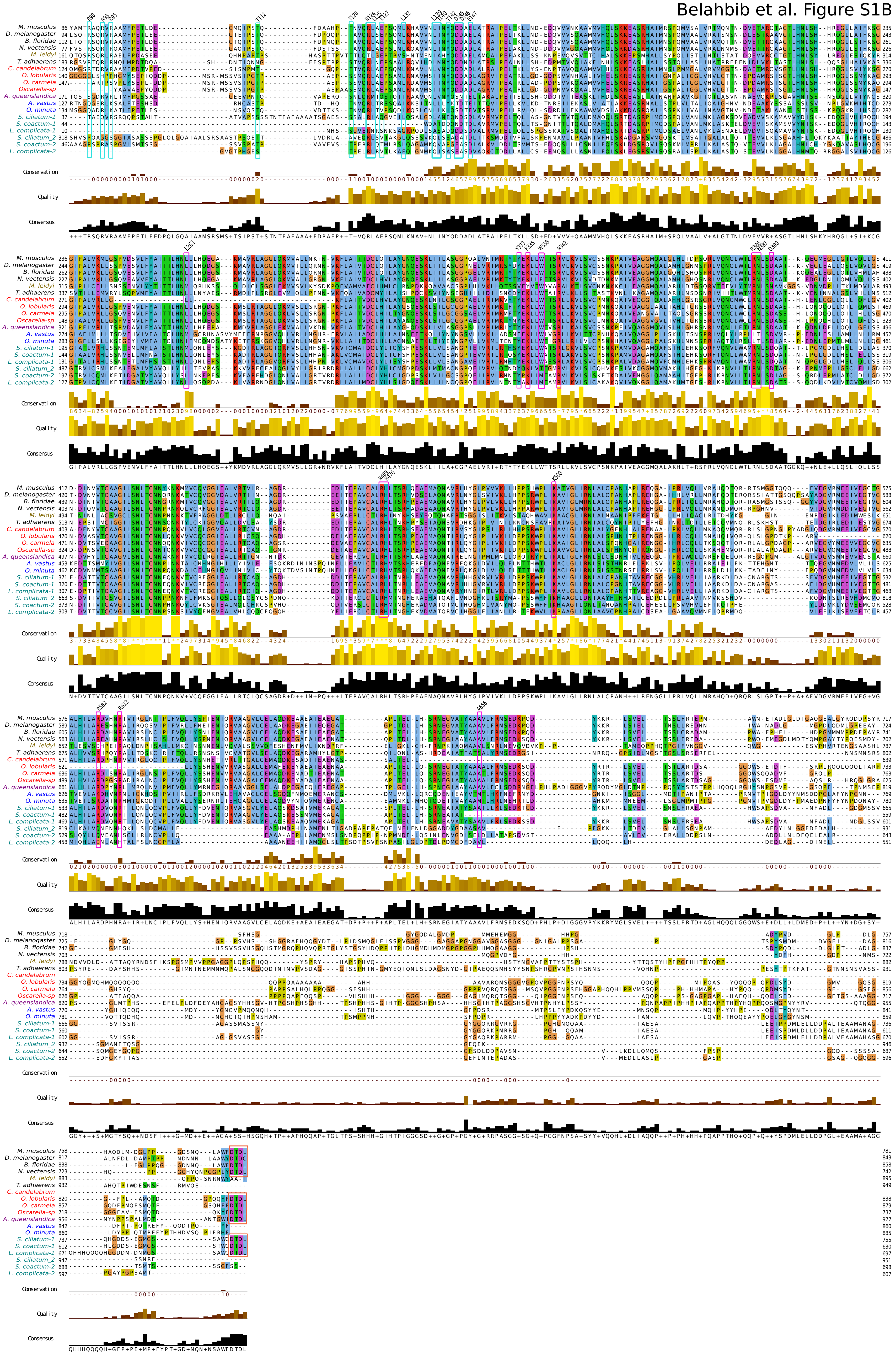

Belahbib et al. Figure S1C

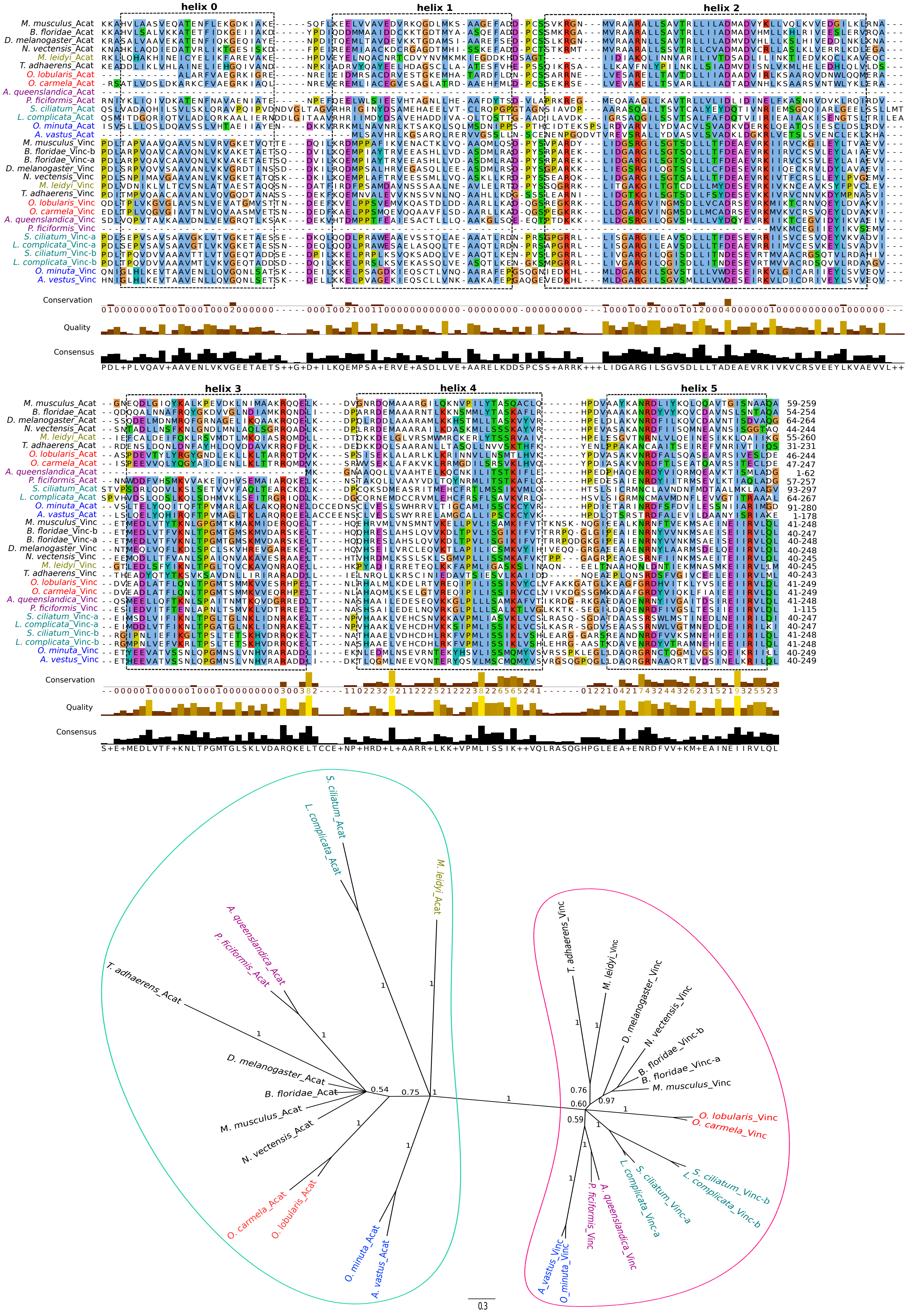

Belahbib et al. Figure S2

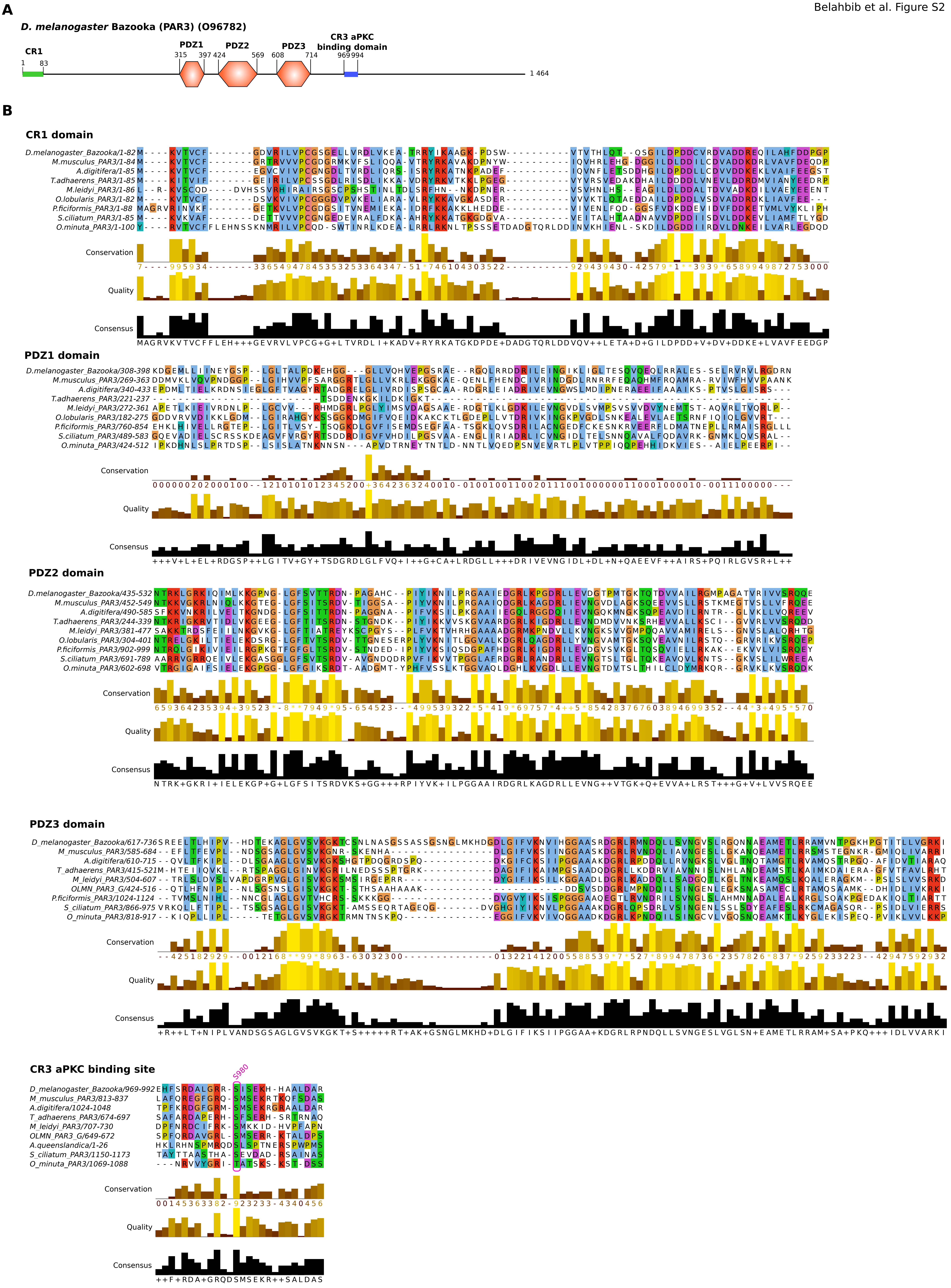

Belahbib et al. Figure S5

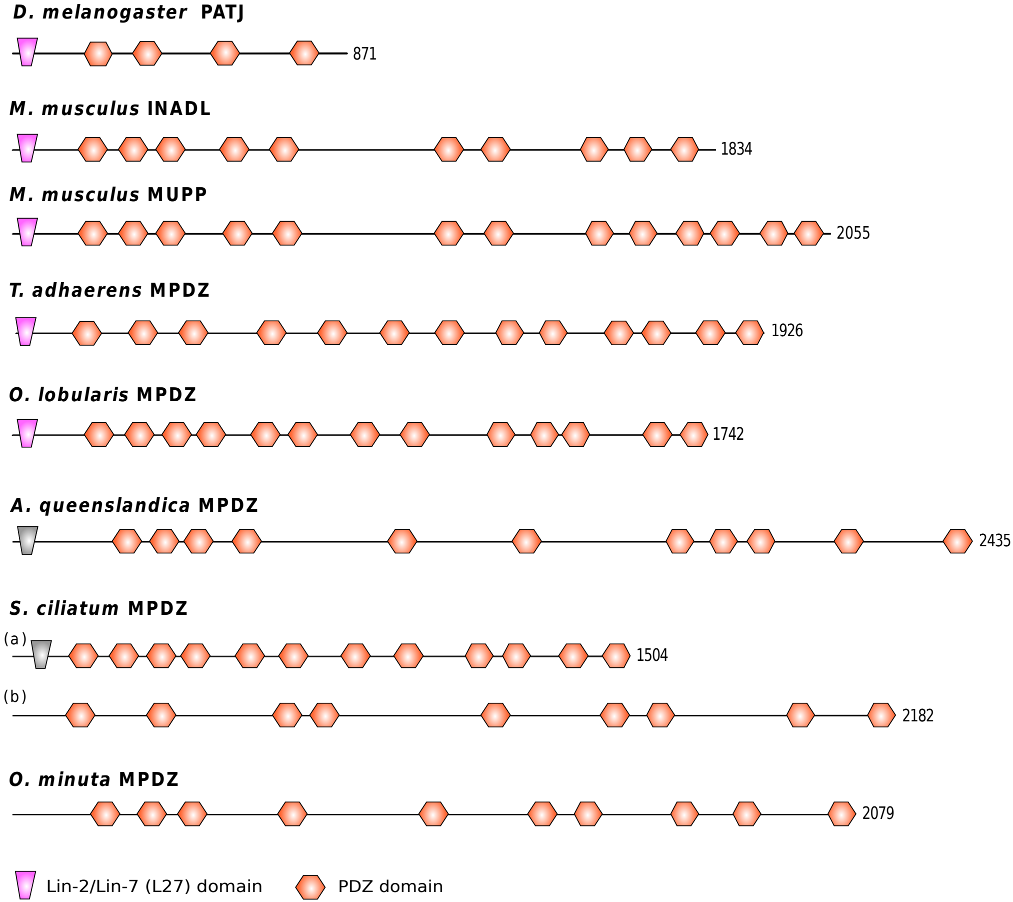

Table S1. Domain structures of the Lethal giant larvae (LGL) proteins in various metazoans
SMART domain diagram of *M. musculus* LGL1 (Q80Y17) canonical domain structure, and number of protein domains WD40, LLGL: Lethal giant larvae homologue 2 (Interpro domain IPR013577) and Lgl_C: Lethal giant larvae(Lgl) like, C-terminal (Pfam PF08596) for *M. musculus* (M. m); *Drosophila melanogaster* (D. m); *Nematostella vectensis* (N. v); *Oscarella lobularis* (O. l); *Oopsacas minuta* (O. m); *Sycon ciliatum* (S. c); *Amphimedon queenslandica* (A. q); *Trichoplax adhaerens* (T. a) and *Mnemiopsis leidyi* (M. l)

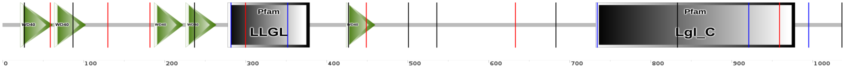

	M. m	D. m	N. v	O. l	O. m	S. c	A. q		T. a	M. l
WD40	5	4	5	6	5	6	5	4	6	4
LLGL	1	1	1	1	1	1	1	1	1	1
Lgl_C	1	1	1	1	1	1	1	1	1	1
Length in aa	1, 036	1, 161	920	1, 117	1, 032	1, 241	1169<br>1130		1093	1170

Table S2. Domain structures of the Scribble (Src) proteins in various metazoans

SMART domain diagram of *M. musculus* Scribble (Q80U72) canonical domain structure, and number of protein domains LRR (leucine rich repeats), LRR TYP (leucine rich repeats, typical) and PDZ for *M. musculus* (M. m); *Drosophila melanogaster* (D. m); *Nematostella vectensis* (N. v); *Oscarella lobularis* (O. l); *Oopsacas minuta* (O. m); *Sycon ciliatum* (S. c); *Amphimedon queenslandica* (A. q); *Trichoplax adhaerens* (T. a) and *Mnemiopsis leidyi* (M. l).

M. leidyi possesses multiple proteins containing LRR domains but since none PDZ domain is associated, these proteins were not taken into acount.

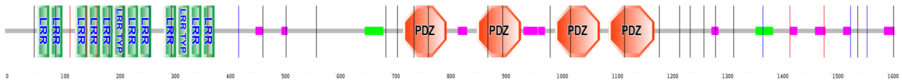

	M. m	D. m	N. v	O. l	O. m	S. c	A. q	T. a
LRR	10	14	12	12	11	13	10	12
LRR TYP	2	0	0	0	2	0	0	0
PDZ	4	4	4	3	3	3	3	4
Length in aa	1, 612	1, 756	1, 030	1, 460	978	1, 011	1, 561	1, 145

Table S3 :characteristics of the new private databases used in this study

	Taxa		species	Dataset	raw reads	corrected/selected reads	mapped reads	depth
**Porifera**	Hexactinellida	Leucopsacidae	*Oopsacas minuta*	Pacbio	751 460	124 793	112 130	
				PE [1]	395 851 746	335 361 472	234 150 237	380
				MP[2]_3-5kb	245 157 912	92 657 868	24 712 816	54
				MP_5-8kb	274 964 868	116 755 522	25 643 839	56
				MP_8-11kb	191 742 544	79 280 536	15 423 954	34
				Transcriptome	421 126 244		207 529 788	1 443
	Homoscleromorpha	Oscarellidae	*Oscarella lobularis*	PE	307 384 094	112 751 740	51 225 276	97
				MP_3-5kb	254 563 152	106 866 702	20 327 965	51
				MP_5-8kb	251 314 628	106 866 702	18 165 914	45
				MP_8-11kb	295 517 160	114 050 246	13 598 285	34
				Transcriptome	380 096 086		231 475 388	710

1: Paired end
2: Mate Pair

Table S4: public databases used in this study : nature (genome/transcriptome) and link for accession

	Taxa	Species	Database name	Sequence Type	URL
Porifera	Demospongiae	*Amphimedon queenslandica*	Amphimedon_queenslandica.Aqu1.30.dna.genome.fa	Genome	ftp://ftp.ensemblgenomes.org/pub/metazoa/release-30/fasta/amphimedon_queenslandica/dna/
			Aqu2_Genes_proteins.fasta	Prediction 1	http://amphimedon.qcloud.qcif.edu.au/downloads.html
			Aqu2.1_Genes_proteins.fasta	Prediction 2	
			Aqu2.1_Genes_mRNA.fasta	Transcriptome	
		Petrosia ficiformis	Petrosia_ficiformis_CLC.fasta	Transcriptome	https://dataverse.harvard.edu/dataset.xhtml?persistentId=doi:10.7910/DVN/24737
	Hexactinellida	*Aphrocallistes vastus*	Aphrocallistes_transcriptome-alberta.fasta	Transcriptome	https://era.library.ualberta.ca/files/bvd66w001v#.WEbM_tFVKlM
	Calcarea	*Sycon ciliatum*	SCIL_WGA_130802	Genome	http://www.compagen.org/datasets.html
			SCIL_P-CDS_130802	Prediction	
			SCIL_T-CDS_130802	Transcriptome 1	
		Sycon coactum	Sycon_transcriptome-alberta.fasta	Transcriptome 2	https://era.library.ualberta.ca/files/bjh343s467
		Leucosolenia complicata	LCOM_T-CDS_130802	Transcriptome	http://www.compagen.org/datasets.html
	Homoscleromorpha	*Oscarella sp.*	Osp._T-CDS_120614	Transcriptome	http://www.compagen.org/datasets.html
		Oscarella carmela	OCAR_WGA_120614.fas	Genome	
			OCAR_T-CDS_130911	Transcriptome	
		Corticium candelabrum	Corticium_candelabrum_CLC.fasta	Transcriptome	https://dataverse.harvard.edu/dataset.xhtml?persistentId=doi:10.7910/DVN/24737
Cnidaria	Anthozoa	*Nematostella vectensis*	Nematostella_vectensis.GCA_000209225.1.30.dna.genome.fa	Genome	ftp://ftp.ensemblgenomes.org/pub/metazoa/release-30/fasta/nematostella_vectensis/dna/
			Nematostella_vectensis.GCA_000209225.1.30.pep.genome.fa	Prediction	ftp://ftp.ensemblgenomes.org/pub/metazoa/release-30/fasta/nematostella_vectensis/pep/
		Acropora digitifera	GCA_000222465.1_Adig_1.0_genomic.fna	Genome	ftp://ftp.ncbi.nlm.nih.gov/genomes/all/GCA/000/222/465/GCA_000222465.1_Adig_1.0/
			adi_transcriptome_assembly.v1.fa	Transcriptome	http://marinegenomics.oist.jp/coral/viewer/download?project_id=3
Placozoa		*Trichoplax adhaerens*	Trichoplax_adhaerens.ASM15027v1.30.dna.genome.fa	Genome	ftp://ftp.ensemblgenomes.org/pub/metazoa/release-30/fasta/trichoplax_adhaerens/dna/
			Trichoplax_adhaerens.ASM15027v1.30.pep.all.fa	Prediction	ftp://ftp.ensemblgenomes.org/pub/metazoa/release-30/fasta/trichoplax_adhaerens/pep/
Ctenophora	Lobata	*Mnemiopsis leidyi*	Mnemiopsis_leidyi.GCA_000226015.1.30.dna.genome.fa	Genome	ftp://ftp.ensemblgenomes.org/pub/metazoa/release-30/fasta/mnemiopsis_leidyi/dna/
			Mnemiopsis_leidyi.GCA_000226015.1.dna.nonchromosomal.fa	Genome	ftp://ftp.ensemblgenomes.org/pub/metazoa/release-33/fasta/mnemiopsis_leidyi/dna/
			Mnemiopsis_leidyi.GCA_000226015.1.30.pep.all.fa	Prediction	ftp://ftp.ensemblgenomes.org/pub/metazoa/release-33/fasta/mnemiopsis_leidyi/pep/
			Ml_Trinity_transcripts.fa	Transcriptome 1	https://kona.nhgri.nih.gov/mnemiopsis/download/download.cgi?dl=transcript
			Mnemiopsis_leidyi_Illumina_RNA-seq	Transcriptome 2	http://neurobase.rc.ufl.edu/pleurobrachia/download
	Pleurobrachiidae	*Pleurobrachia bachei*	GCA_000695325.1_P.bachei_draft_genome_v.1.1_genomic.fna	Genome 1	ftp://ftp.ncbi.nlm.nih.gov/genomes/all/GCA/000/695/325/GCA_000695325.1_P.bachei_draft_genome_v.1.1/
			genome.v1b.fa	Genome 2	http://rogaevlab.ru/pleurobrachia/data/genome.v1b.fa
			Pleurobrachia_bachei_transcriptome_assembly.fasta	Transcriptome	http://rogaevlab.ru/pleurobrachia/data/Pleurobrachia_bachei_transcriptome_assembly.fasta.gz
	Beroida	*Beroe abyssicola*	Beroe_abyssicola_Illumina_RNA-seq	Transcriptome	http://neurobase.rc.ufl.edu/pleurobrachia/download
		Beroe sp	Beroe_sp_pink_Illumina_RNA-seq	Transcriptome	
Mollusca	Gastropoda	*Aplysia californica*	acl_ref_AplCal3.0_chrUn.fa	Genome	ftp://ftp.ncbi.nlm.nih.gov/genomes/Aplysia_californica/CHR_Un/
Chordata	Cephalochordata	*Branchiostoma floridae*	Branchiostoma_floridae_v2.0.assembly.fasta	Genome	http://genome.jgi.doe.gov/Brafl1/Brafl1.home.html
			transcripts.Brafl1.fasta (assembly 1.0)	Transcriptome	

Table S5: Cadherin-Catenin Complex (CCC) sequence retrieval: Accession numbers or contig/scaffold references where candidate genes
In bold accession numbers of sequences annotated from our two new transcriptomic and genomic sponge datasets.
1: see table S4 for the links to corresponding data sources; 2: Prediction of the protein sequence and/or the function

Complex	Gene	Taxa	Species	Sequence ID	Data sources[1] for prediction[2]
			Mus muscul	P09803	
		Chordata	*Gallus gallu*	P08641	
			Ciona intest	XP_009859529.1	
		Arthropoda	*Drosophila r*	Q24298	
		Cnidaria	*Nematostell*	XP_001631293.1	
		Placozoa	*Trichoplax adhaerens*		Scaffold_4 (Genome)
			Oscarella k	**MF780952**	
	E-cadherin		*Oscarella sp*	G4W9D8	comp20092_c0_seq1
			Amphimedon queenslandica		Aqu2.16910_001 (Transcriptome 1)
			Petrosia ficiformis		SpoMult2_CAGATC_L001_R1_001_contig_5633
		Porifera	*Sycon ciliatum*		scpid4789 (prediction)
			Sycon coactum		Contig_12142 (Transcriptome)
			Leucosolenia complicata		lctid26665 (Transcriptome)
			Oopsacas r	**MF959449**	
			Aphrocallites vastus		comp20072_c0_seq1 (Transcriptome)
		Ctenophora	*Mnemiopsis leidyi*		ML00359a-PA (prediction)
		Chordata	*Mus muscul*	Q02248	
			Branchiosto	Q4U478	
		Arthropoda	*Drosophila r*	P18824	
		Cnidaria	*Nematostella vectensis*		NEMVEscaffold_183 (Genome)
		Placozoa	*Trichoplax adhaerens*		TriadP22780 (prediction)
		Porifera	*Corticium candelabrum*		Contig_16639 (Transcriptome)
			Oscarella k	**MF780953**	
			Oscarella carmela		comp41806_c0_seq1 (Transcriptome)
	Beta-catenin		*Oscarella sp*	G4W9D9	comp3163_c0_seq1 (Transcriptome)
			Sycon ciliatu	A0A077SQP5 and A0A077SQX3	
			Sycon coactum		Contig_5031 (transcriptme) and Contig_19 (Transcriptome)
			Leucosolenia complicata		lctid45409 and lctid54960
			Amphimedo	NP_001266234.1/E2IJA6	Aqu2.23648 (Transcriptome 2)
			Oopsacas r	**MF959459**	
			Aphrocallites vastus		comp17679_c0_seq1 (Transcriptome)
		Ctenophora	*Mnemiopsis*	E3UKC7_MNELE	ML073715a-RA (prediction)
		Chordata	*Mus muscul*	P30999	
			Branchiostoma floridae		Bf_V2_15 (Transcriptome 1)
		Arthropoda	*Drosophila r*	Q7PLI0	
		Cnidaria	*Acropora di*		adi_EST_assem_414 (Transcriptome)
					adi_EST_assem_2325 (Transcriptome)
		Placozoa	*Trichoplax adhaerens*		Scaffold_11 (Genome)
			Oscarella k	**MF780954**	
	ta-catenin/P		*Oscarella sp. SN-2011*		comp5428_c1_seq1 (Transcriptome)
			Oscarella carmela		comp43311_c0_seq1 (Transcriptome)
		Porifera	***Oopsacas r***	**MF959460**	
			Leucosolenia complicata		lctid32410 (Transcriptome)
			Sycon ciliatum		sctid48573 (Transcriptome)
			Sycon coactum		Contig_12039 (Transcriptome)
		Ctenophora	*Mnemiopsis leidyi*		ML002622a-PA (prediction)
		Chordata	*Mus muscul*	P26231	
			Branchiosto	C3Y5U8	
		Arthropoda	*Drosophila r*	P35220	
		Cnidaria	*Nematostella vectensis*		EDO48695 (prediction)
		Placozoa	*Trichoplax a*	B3S2X1	TriadP28149 (prediction)
			Oscarella k	**MF780955**	
	lpha-catenin		*Oscarella carmela*		comp32633_c0_seq8 (prediction)
			Amphimedon queenslandica		Aqu2.37246_001 (Transcriptome)
		Porifera	*Petrosia ficiformis*		Contig_18097 + contig_10589
			Sycon ciliatum		sctid49136 (Transcriptome)
			Leucosolenia complicata		lctid42595 (Transcriptome)
			Oopsacas r	**MF959461**	
			Aphrocallites vastus		comp19808_c1_seq1+comp20080_c0_seq3 (Transcriptome)
		Ctenophora	*Mnemiopsis leidyi*		ML02753a (prediction)
		Chordata	*Mus muscul*	Q64727	
			Branchiostoma floridae		280829 (Transcriptome 2) and 80007 (Transcriptome 2)
		Arthropoda	*Drosophila r*	O46037	
		Cnidaria	*Nematostella vectensis*		EDO35762 (prediction)
		Placozoa	*Trichoplax adhaerens*		TriadP52110 (prediction)
			Oscarella k	**MG003328**	**c12786_g1_i1 (Transcriptome trinity)**
	Vinculin		*Oscarella carmela*		comp41876_c0_seq1 (Transcriptome)
			Amphimedon queenslandica		Aqu2.1.44113_001 (Transcriptome)
		Porifera	*Petrosia ficiformis*		contig_687 (Transcriptome)
			Sycon ciliatum		stpid41737 (Transcriptome) and stpid35434 (Transcriptome)
			Leucosolenia complicata		lctid48881 (Transcriptome) and lctid7225 (Transcriptome)
			Oopsacas r	**MF959462**	
			Aphrocallites vastus		comp21341_c0_seq8 (Transcriptome)
		Ctenophora	*Mnemiopsis leidyi*		ML148910a (prediction)

Table S6: Polarity complex sequence retrieval: Accession numbers or contig/scaffold references where candidate genes were identified. Sequences from our new genomic and transcriptomic datasets are in bold. 1: links to corresponding data sources are provided in table S4; 2: Prediction of the protein sequence and/or the function; 3: names used in figure

Complex	Gene	Taxa	Species	copy name[3]	Sequence ID	Data sources[1] for prediction[2]	NCBI links for new sequences
		**Chordata**	*Mus musculus*		Q80Y17		
		**Arthropoda**	*Drosophila melanogaster*		P08111		
		**Cnidaria**	*Nematostella vectensis*			EDO48036 (protein translation)	
		**Placozoa**	*Trichoplax adhaerens*			Scaffold_16 (genome) and TriadP31093 (prediction)	
			Oscarella lobularis		**MF780964**		https://www.ncbi.nlm.nih.gov/nuccore/MF780964
			Oscarella carmela			comp39237_c0_seq14	
	**LGL**		***Oopsacas minuta***		**MF959464**		https://www.ncbi.nlm.nih.gov/nuccore/MF959464
		**Porifera**	*Aphrocallites vastus*			comp6849_c0_seq1	
			Sycon ciliatum			sctid17182	
			Leucosolenia complicata			lctid21810	
			Amphimedon queenslandica			Aqu2.1.23963_001 (LGL1; transcriptome) and Aqu2.1.35936_001 (LGL2; transcriptome)	
			Petrosia ficiformis			Contig_4228 (LGL1; transcriptome) and Contig_10319 (LGL2; transcriptome)	
		**Ctenophora**	*Mnemiopsis leidyi*			ML24811a-PA (prediction)	
		**Chordata**	*Mus musculus*		Q80U72		
		**Arthropoda**	*Drosophila melanogaster*		AAF26357.2		
		**Cnidaria**	*Nematostella vectensis*		A7SS78	EDO33432 (protein translation)	
		**Placozoa**	*Trichoplax adhaerens*			scaffold_9 (genome)	
			Oscarella lobularis		**MF780962**		https://www.ncbi.nlm.nih.gov/nuccore/MF780962
**SCR**	**Scribble**		*Oscarella carmela*			comp42638_c0_seq1 (transcriptome)	
			Oopsacas minuta		**MF959465**		https://www.ncbi.nlm.nih.gov/nuccore/MF959465
		**Porifera**	*Aphrocallites vastus*			comp22172_c0_seq3 (transcriptome)	
			Sycon ciliatum			Sctid30593 (transcriptome)	
			Amphimedon queenslandica		XP_019852372.1	Aqu2.1.31180_001 (transcriptome 2)	
			Petrosia ficiformis			Contig_3969	
			Homo sapiens	H.sapiens_DLG1	Q12959		
		**Chordata**	*Mus musculus*	M.musculus_DLG1	Q811D0		
			Branchiostoma floridae	B.floridae_DLG		Bf_V2_147 (genome)	
		**Arthropoda**	*Drosophila melanogaster*	D.melanogaster_DLG1	P31007		
		**Cnidaria**	*Nematostella vectensis*	N.vectensis_DLG	A7SFE0	EDO37538 (protein translation)	
		**Placozoa**	*Trichoplax adhaerens*	T.adhaerens_DLG	B3RLX1	TriadP63520 (prediction)	
			Amphimedon queenslandica	A.queenslandica_DLG		Aqu2.1.42154_001+Aqu2.1.05916_001 (transcriptome)	
	**DLG**		***Oscarella lobularis***	**O.lobularis_DLG**	**MF780963**		https://www.ncbi.nlm.nih.gov/nuccore/MF780963
			Oscarella carmella	O.carmela_DLG		comp41736_c0_seq1 (transcriptome)	
			Oopsacas minuta	**O.minuta_DLG**	**MF959455**		https://www.ncbi.nlm.nih.gov/nuccore/MF959455
		**Porifera**	*Aphrocalistes vastus*	A.vastus_DLG		comp20644_c0_seq1 (transcriptome)	
			Sycon ciliatum	S.ciliatum_DLGa		scpid36247 (prediction)	
				S.ciliatum_DLGb		scpid32996 (prediction)	
			Leusosolenia complicata	L.complicata_DLGa		lctid29073 (transcriptome)	
				L.complicata_DLGb		lctid25548 (transcriptome)	
		**Ctenophora**	*Mnemiopsis leidyi*	M.leidyi_DLG		comp19374_c0_seq2 (transcriptome 1)	

CRUMBS

Crumbs	Chordata	*Homo sapiens*	Human_CRUM1	NP_001180569.1		
			Human_CRUM2	NP_775960.4		
			Human_CRUM3	NP_631900.1		
		Pan troglodytes	Chimpanzee_Crb1	XP_009438564.1		
			Chimpanzee_Crb2	XP_528426.3		
		Macaca mulatta	Macaca_Crb1	H9FS33		
			Macaca_Crb2	H9F357		
			Macaca_Crb3	H9EY01		
		Mus musculus	Mouse_CRUM1	NP_573502.2		
			Mouse_CRUM2	NP_001157038.1		
			Mouse_CRUM3	NP_808306.1		
		Bos taurus	Caw_Crb1	DAA21022.1		
			Caw_Crb2	XP_003586718.1		
		Canis lupus familiaris	Dog_Crb1	XP_005622350.1		
		Monodelphis domestica	Opossum_Crb1	XP_007481075.1		
			Opossum_Crb2	XP_007474690.1		
		Takifugu rubripes	Takifugu_Crb1	XP_003974391.1		
			Takifugu_Crb2	XP_011617710.1		
		Danio rerio	Danio_Crb1	NP_001038408.1		
			Danio_Crb2	NP_001038764.1		
			Danio_Crb3	NP_001038787.1		
		Gallus gallus	Gallus_Crb1_iso1	XP_003641718.2		
			Gallus_Crb2_iso1	XP_004946066.1		
		Xenopus (Silurana) tropicalis	Xenopus_Crb1	XP_002932223.2		
			Xenopus_Crb2	XP_004918981.1		
			Xenopus_Crb3	NP_001012706.1		
		Saccoglossus kowalevskii	Saccoglossus_CrbL	XP_006817326.1		
	Echinodermata	*Strongylocentrotus purpuratus*	Urchin_Crb1_iso2	XP_001188090.2		
	Arthropoda	*Anopheles darlingi*	Anopheles_Crb	ETN63846.1		
		Apis cerana	Apis_Crb_iso1	XP_016917411.1		
		Drosophila melanogaster	Fruitfly_CrbB	NP_001036751.1		
		Tribolium castaneum		XP_970640.2		
		Nasonia vitripennis	Nasonia_Crb-iso1	XP_016838844.1		
		Daphnia magna	Daphnia_Crb	JAM66283.1		
	Nematoda	*Caenorhabditis elegans*	C.elegans_CRB1	NP_510822.1		
			C.elegans_CRB3	NP_001041224.1		
	Cnidaria	*Nematostella vectensis*	Nematostella_CrbA		NEMVEscaffold_237 (genome)	
			Nematostella_CrbB		NEMVEscaffold_217 (genome)	
		Trichoplax adhaerens	Trichoplax_CrbA		Scaffold_2 (genome)	
			Trichoplax_CrbB			
	Porifera	*Amphimedon queenslandica*		I1FTA5 (uncharacterized)	Aqu2.28529_001 or Aqu2.1.31775_001 (transcriptome)	
		Sycon ciliatum			scis1578 (genome)	
		Oscarella lobularis		**MF780959**		https://www.ncbi.nlm.nih.gov/nuccore/MF780959
		Oopsacas minuta		**MF959458**		https://www.ncbi.nlm.nih.gov/nuccore/MF959458
		Aphrocallistes vastus			comp20814_c0_seq1 (transcriptome)	
MPP5/Pals1/Stardust	Chordata	*Homo sapiens*	CSKP_H.sapiens	AAB88198.1		
			MPP2_H.sapiens	NP_001265299.1		
			MPP3_H.sapiens	AAH56865.1		
			MPP4_H.sapiens	NP_149055.1		
			MPP5_H.sapiens	XP_011535389.1		
			MPP6_H.sapiens	AAH23638.1		
			MPP7_H.sapiens	AAH38105.1		
		Mus musculus	CSKP_M.musculus	NP_001271432.1		
			MPP2_M.musculus	NP_057904.1		
			MPP3_M.musculus	NP_031889.2		
			MPP4_M.musculus	NP_001158154.1		
			MPP5_M.musculus	NP_062525.1		
			MPP6_M.musculus	AAI45365.1		
			MPP7_M.musculus	AAI18059.1		
		Branchiostoma floridae	CSKP_B.floridae	C3Y7R3 (uncharacterized)	jgi|Brafl1|119718|estExt_fgenesh2_pg.C_330113 (transcriptome)	
			MPP2-6_B.floridae	C3Z0H4 (uncharacterized)	jgi|Brafl1|265353|estExt_GenewiseH_1.C_880038 (transcriptome)	
			MPP3-4-7_B.floridae	C3Z0H2 (uncharacterized)	jgi|Brafl1|79765|fgenesh2_pg.scaffold_88000029 (transcriptome)	
			MPP5_B.floridae	C3Y0J3 (uncharacterized)	jgi|Brafl1|60486|fgenesh2_pm.scaffold_231000005 (transcriptome)	
	Arthropoda	*Drosophila melanogaster*	CSKP_D.melanogaster	NP_732661.1		
			Mena3_D.melanogaster	NP_610642.2		
			Stardust_D.melanogaster	NP_996375.1		
	Cnidaria	*Nematostella vectensis*	CSKP_N.vectensis	A7SAA1	EDO39364 (protein translation)	
			MPP2-6_N.vectensis	A7SFE0 (incomplete sequence)	NEMVEscaffold_138 (complete sequence from genome)	
			MPP5_N.vectensis		NEMVEscaffold_261 (genome)	
			MPP_N.vectensis	A7RMI8	EDO47352 (Prediction)	
		Acropora digitifera	MPP2-6_A.digitifera		adi_EST_assem_5910 (transcriptome)	
			MPP5_A.digitifera		adi_EST_assem_1774 (transcriptome)	
	Placozoa	*Trichoplax adhaerens*	CSKP_T.adhaerens		TriadP55243 (prediction)	
			CSKP_T.adhaerens		TriadP63748 (prediction)	
			MPP_T.adhaerens		TriadP54413 (prediction)	
			MPP5_T.adhaerens		TriadP52734 (prediction)	
		Oscarella lobularis	**MP2-6_O.lobularis**	**MF780968**		https://www.ncbi.nlm.nih.gov/nuccore/MF780968
			**MPP_O.lobularis**	**MF780969**		https://www.ncbi.nlm.nih.gov/nuccore/MF780969
			**MPP5_O.lobularis**	**MF780960**		https://www.ncbi.nlm.nih.gov/nuccore/MF780960
		Oscarella carmela	MPP2-6_O.carmela		comp42953_c0_seq1 (transcriptome)	
			MPP_O.carmela		comp14312_c0_seq1 (transcriptome)	
			MPP5_O.carmela		comp2196_c0_seq1 (transcriptome)	

	Porifera	*Amphimedon queenslandica*	MPP-CASK_A.queenslandica	UPI00021A50C0	Aqu2.23933_001 (transcriptome (1))	
			MPP_A.queenslandica		Aqu2.23934_001 (transcriptome (1))	
			MPP5_A.queenslandica		Aqu2.33970_001 (transcriptome (1))	
		Petrosia ficiformis	MPP-CASK_P.ficiformis		Contig_400 (Transcriptome)	
			MPP5_P.ficiformis		Contig_945 (Transcriptome)	
		Oopsacas minuta	**MPP2-6-CSKP_O.minuta**	**MG003331**	c18256_g1_i3 (transcriptome)	https://www.ncbi.nlm.nih.gov/nuccore/MG003331
			**MPP5/Stardust.O,minuta**	**MG003330**	c19478_g1_i1 (transcriptome)	https://www.ncbi.nlm.nih.gov/nuccore/MG003330
		Aphrocallites vastus	MPP2-6_A.vastus		comp30003_c0_seq1 (transcriptome)	
			MPP_A.vastus		comp17674_c0_seq1 (transcriptome)	
		Sycon ciliatum	MPP2-6_S.ciliatum-65043		sctid65043 (transcriptome)	
			MPP2-6_S.ciliatum-51990		sctid51990 (transcriptome)	
			MPP_S.ciliatum		sctid31293 (transcriptome)	
			MPP5_S.ciliatum		sctid19242 (transcriptome)	
		Leucosolenia complicata	MPP2-6_L.complicata		lctid72183 (transcriptome)	
			MPP_L.copmplicata		lctid53457 (transcriptome)	
			MPP5_L.complicata		lctid20340 (transcriptome)	
	Ctenophora	*Mnemiopsis leidyi*	MPP_2-6_M.leidyi-120734		ML120734a-PA (prediction)	
			MPP_2-6_M.leidyi-35887		ML35887a-PA (prediction)	
			MPPa_M.leidyi-096814		ML096814a-PA (prediction)	
			MPPb_M.leidyi-305528		ML305528a-PA (prediction)	
PATJ/MPDZ	Chordata	*Homo sapiens*	*H.sapiens_INADL*	NP_795352.2		
			H.sapiens_MPDZ	NP_001248335.1		
		Mus musculus	*M.musculus_INADL*	NP_001005784.1		
			M.musculus_MPDZ	NP_001292213.1		
	Arthropoda	*Drosophila melanogaster*	*D.melanogaster_INAD*	NP_726260.1		
	Cnidaria	*Nematostella vectensis*	*N.vectensis_PATJ*	A7SRG3_NEMVE	EDO33706 (prediction)	
	Placozoa	*Trichoplax adhaerens*	*T.adhaerens_PATJ*	B3S1C8	TriadP27973 (prediction)	
	Porifera	*Amphimedon queenslandica*	*A.queenslandicA.MUPP-PATJ*	1FDE9	Aqu2.1.24128_001	
		Petrosia ficiformis	*P.ficiformis_MUPP-PATJ*		contig_23858 + contig_6749 (transcriptome)	
		Oscarella lobularis	***O.lobularis_MUPP-PATJ***	**MF780961**		https://www.ncbi.nlm.nih.gov/nuccore/MF780961
		Oopsacas minuta	***O.minuta_MPDZ***	**MF959457**		https://www.ncbi.nlm.nih.gov/nuccore/MF959457
		Aphrocallites vastus	A.vastus_MPDZ		comp26018_c0_seq1 (transcriptome)	
		Sycon ciliatum	S.ciliatum_MPDZ-a		sctid12690 (transcriptome)	
			S.ciliatum_MPDZ-b		sctid4360 (transcriptome)	
		Leucosolenia complicata	L.complicata_MPDZ-a		lctid18737 (transcriptome)	
			L.complicata_MPDZ-b		lctid8806 (transcriptome)	
LIN	Chordata	*Homo sapiens*	H.sapiens_LIN7A	O14910		
			H.sapiens_LIN7B	Q9HAP6		
			H.sapiens_LIN7C	Q9NUP9		
		Mus musculus	M.musculus_LIN7A	Q8JZS0		
			M.musculus_LIN7B	O88951		
			M.musculus_LIN7C	O88952		
		Branchiostoma floridae	B.floridae_LIN		jgi|Brafl1|288594|estExt_gwp.C_6290018 (transcriptome)	
	Cnidaria	*Nematostella vectensis*	N.vectensis_LIN	XP_001639785.1 (predicted protein)	EDO47722 (prediction)	
	Placozoa	*Trichoplax adhaerens*	T.adhaerens_LIN	XP_002117602.1 (hypothetical protein)	TriadP61629 (prediction)	
	Porifera	*Amphimedon queenslandica*	A.queenslandica_LIN	XP_019856715.1	Aqu2.19247_001 (transcriptome)	
		Oscarella lobularis	**O.lobularis_LIN**	**MG003329**	c12448_g1_i1 (transcriptome Trinity)	https://www.ncbi.nlm.nih.gov/nuccore/MG003329
		Oscarella carmela	O.carmela_LIN		comp25532_c0_seq1 (transcriptome)	
		Oopsacas minuta	**O.minuta_LIN**	**MF959456**		https://www.ncbi.nlm.nih.gov/nuccore/MF959456
		Aphrocallites vastus	A.vastus_LIN		comp25879_c0_seq1 (transcriptome)	
		Sycon ciliatum	S.ciliatum_LIN		lctid35214 (transcriptome)	
		Leucosolenia complicata	L.complicata_LIN		lctid40490 (transcriptome)	
	Ctenophora	*Mnemiopsis leidyi*	M.leidyi_LIN		ML05292a-PA (prediction)	

PAR	PAR6	Chordata	*Mus musculus*		Q9Z101		
					Q9JK83		
			Homo sapiens		Q9NPB6		
		Arthropoda	*Drosophila melanogaster*		NP_573238.1		
		Cnidaria	*Nematostella vectensis*			Scaffold_196 (Genome)	
		Placozoa	*Trichoplax adhaerens*			ML3517 (Genome)	
		Ctenophora	*Mnemiopsis leidyi*			Scaffold_1 (Genome)	
		Porifera	***Oscarella lobularis***		**MF780956**		https://www.ncbi.nlm.nih.gov/nuccore/MF780956
			Oscarella carmela			comp11890_c0_seq1 (transcriptome)	
			Oopsacas minuta		**MF959450**		https://www.ncbi.nlm.nih.gov/nuccore/MF959450
			Aphrocallites vastus			comp4505_c0_seq1 (transcriptome)	
			Sycon ciliatum			sctid75477 (transcriptome)	
			Leucosolenia complicata			lctid87073 (transcriptome)	
			Amphimedon queenslandica			Aqu2.34456_001 (transcriptome 1)	
			Petrosia ficiformis			Contig_11878 (transcriptome)	
	PAR3	Chordata	*Mus musculus*		Q99NH2		
		Arthropoda	*Drosophila melanogaster*		O96782		
		Cnidaria	*Nematostella vectensis*			scaffold_26 (Genome)	
		Placozoa	*Trichoplax adhaerens*			TriadP55106 (prediction)	
		Ctenophora	*Mnemiopsis leidyi*			ML006311a-PA (prediction)	
		Porifera	***Oscarella lobularis***		**MF780957**		https://www.ncbi.nlm.nih.gov/nuccore/MF780957
			Amphimedon queenslandica			contig13508 (Genome)	
			Petrosia ficiformis			contig_4639 (transcriptome)	
			Sycon ciliatum			scpid12913 (prediction)	
			Oopsacas minuta		**MF959463**		https://www.ncbi.nlm.nih.gov/nuccore/MF959463
	aPKC	Chordata	*Mus musculus*	zeta	Q02956		
			Danio rerio		Q4JG04		
			Mus musculus	iota	Q62074		
			Danio rerio		Q6DI53		
			Mus musculus	lambda	BAA32499.1		
			Danio rerio		AAK91291.1		
		Arthropoda	*Drosophila melanogaster*		A1Z9X0		
		Cnidaria	*Nematostella vectensis*		A7RU22	EDO44938 (prediction)	
		Placozoa	*Trichoplax adhaerens*		B3RZX4	TriadP57608 (prediction)	
		Ctenophora	*Mnemiopsis leidyi*			ML008317a-PA (prediction)	
		Porifera	*Petrosia ficiformis*			contig_14294 (transcriptome)	
			Amphimedon queenslandica			Aqu2.1.41475_001 (Prediction 2)	
			Oopsacas minuta		**MF959451**		https://www.ncbi.nlm.nih.gov/nuccore/MF959451
			Aphrocallistes vastus			comp18698_c0_seq1 (transcriptome)	
			Sycon ciliatum			scpid56290 (prediction)	
			Leucosolenia complicata			lcpid18427 (prediction)	
			Oscarella lobularis		**MF780958**		https://www.ncbi.nlm.nih.gov/nuccore/MF780958
			Oscarella carmela			comp29069_c0_seq1 (transcriptome)	
	PKC α-β-ζ	Chordata	*Mus musculus*	gamma	P63318		
				alpha	P20444		
				beta	P68404		
			Danio rerio		Q7SY24		
		Cnidaria	*Nematostella vectensis*		A7RUL8	EDO44925 (prediction)	
		Placozoa	*Trichoplax adhaerens*		B3RQ26	TriadP20496 (prediction)	
		Ctenophora	*Mnemiopsis leidyi*			ML13931a-PA (prediction)	
		Porifera	*Leucosolenia complicata*			lcpid31219 (prediction)	
			Sycon raphanus		O61224		
			Oscarella lobularis		**MF780965**		https://www.ncbi.nlm.nih.gov/nuccore/MF780965
			Oscarella carmela			comp37413_c0_seq29 (transcriptome)	
			Amphimedon queenslandica			Aqu2.1.43329_001 (Prediction 2)	
			Petrosia ficiformis			contig_3863 (transcriptome)	
			Suberites domuncula		O62567		
			Geodia cydonium		O96997		
			Oopsacas minuta		**MF959452**		https://www.ncbi.nlm.nih.gov/nuccore/MF959452
			Aphrocallistes vastus			comp28256_c0_seq1 (transcriptome)	
	PKC δ	Chordata	*Mus musculus*		P28867		
			Danio rerio		Q7ZUC5		
		Porifera	*Leucosolenia complicata*			lcpid5946 (prediction)	
						lcpid32739 (prediction)	
			Sycon ciliatum			scpid34149 (prediction)	
			Sycon raphanus		O61225		
			Oscarella carmela			comp42357_c0_seq1 (transcriptome)	
			Oscarella lobularis		**MF780966**		https://www.ncbi.nlm.nih.gov/nuccore/MF780966
			Suberites domuncula		O62569		
			Petrosia ficiformis			contig_2862 (transcriptome)	
			Amphimedon queenslandica		I1G4K3_AMPQE	Aqu2.1.37442_001 (Prediction 2)	
			Oopsacas minuta		**MF959454**		https://www.ncbi.nlm.nih.gov/nuccore/MF959454
			Aphrocallistes vastus			comp24409_c0_seq1 (transcriptome)	
	PKC ε	Chordata	*Mus musculus*		P16054		
		Arthropoda	*Drosophila melanogaster*		KPC3		
		Cnidaria	*Nematostella vectensis*		A7SL27	EDO35585 (prediction)	
		Placozoa	*Trichoplax adhaerens*		B3S6K6	TriadP30067 (prediction)	
			Oscarella lobularis		**MF780967**		https://www.ncbi.nlm.nih.gov/nuccore/MF780967
			Oscarella carmela			comp17286_c0_seq1 (transcriptome)	
			Sycon ciliatum			scpid30652 (prediction)	

**Porifera**	*Leucosolenia complicata*		lcpid37553 (prediction)	
	Amphimedon queenslandica	I1FUR1_AMPQE	Aqu2.1.32509_001	
	Petrosia ficiformis		contig_3767 (transcriptome)	
	Oopsacas minuta	**MF959453**		https://www.ncbi.nlm.nih.gov/nuccore/MF959453
	Aphrocallistes vastus		comp15198_c0_seq1 (transcriptome)	
